# Powdery mildew resistance prediction in Barley (*Hordeum Vulgare* L) with emphasis on machine learning approaches

**DOI:** 10.1038/s41598-025-02939-3

**Published:** 2025-06-04

**Authors:** Farveh Vahidpour, Hossein Sabouri, Fakhtak Taliei, Sayed Javad Sajadi, Saeed Yarahmadi, Hossein Hosseini Moghaddam

**Affiliations:** 1 Department of Plant Production, Collage of Agriculture Science and Natural Resource, Gonbad Kavous University, Gonbad-E Kavus, 4971799151 Iran; 2 Horticulture-Crops Research Department, Golestan Agricultural and Natural Resources Research and Education Center, Agricultural Research, Education and Extension Organization (AREEO), Gorgan, 4969186951 Iran

**Keywords:** Drought, Salinity, Machine learning, Prediction, Classification, Barley, Agricultural genetics, Machine learning

## Abstract

By employing machine-learning models, this study utilizes agronomical and molecular features to predict powdery mildew disease resistance in Barley (Hordeum Vulgare L). A 130-line F8-F9 barley population caused Badia and Kavir to grow at the Gonbad Kavous University Research Farm on three planting dates (19 November, 19 January, and 19 March), with three replicates in 2018/2019 and 2019/2020. The study employed RReliefF, MRMR, and F-Test feature selection algorithms to identify essential phenotype traits and molecular markers. Subsequently, Decision Tree, Random Forest, Neural Network, and Gaussian Process Regression models were compared using MAE, RMSE, and R2 metrics. The Bayesian algorithm was utilized to optimize the parameters of the machine-learning models. The results indicated that the Neural Network model accurately predicted powdery mildew disease resistance in barley lines. The evaluation based on high R2 values, as well as low MAE and RMSE, highlighted the efficacy of these models in identifying significant phenotype traits and molecular markers associated with disease resistance. The findings demonstrate machine learning models’ potential in accurately predicting powdery mildew disease resistance in Barley. The neural network model specifically showed excellent results in this area because it managed to identify critical phenotypic traits and molecular markers very well. This research highlights the importance of combining AI with molecular markers for improved disease resistance and other desirable crop traits during plant breeding.

## Introduction

Plant disorders caused by harmful bacteria, fungi, viruses, and pests are biological stress. Biological stress is one of the most critical factors affecting Barley (*Hordeum Vulgare L*) yield. Powdery mildew caused by the biotrophic fungus *Blumeria graminis f.sp. hordei* is one of the most destructive and common barley diseases, and achieving sustainable resistance to this disease is one of the main challenges for plant breeders^1^. This disease has gained significant importance recently due to the rapid change in pathotype patterns and agricultural practices^2^. Identifying this disease and determining resistant lines is very important due to the effect of reducing yield and seed quality^3^. Molecular markers provide valuable information for detecting resistance to powdery mildew. The Markers can be used to form geometric maps and study species diversity. Molecular markers can detect genetic differences between organisms and species but cannot remember the target gene^4^. These markers determine the number of genes controlling traits. The abundance of molecular markers on chromosomes is of great help to researchers involved in the analysis and study of plant genomes^5^. The RAPD, SSR, and ISSR markers are widely used in identifying and manipulating the location of genes that control traits. The use of molecular markers with high accuracy speeds up breeding programs, lowers costs, and saves energy, which results in a breakthrough in the analysis of the marker-trait relationship.

The use of machine learning (ML) in predicting the performance of plant products has received attention in recent years^6–11^. The combination of molecular marker information and the predictive capabilities of the ML models will enable researchers to gain better insights into plant genomes, speed up breeding initiatives, and make intelligent moves toward crop improvement strategies^12–15^. The Ml models are advanced modeling techniques employed to predict and analyze genetic values and traits based on the data generated by molecular markers. ML models detect QTL related to soybean quality traits such as protein, oil, and hundred seed weight in soybean genotypes^16^, predicting rice cultivars with low and high CD accumulation with genotypes and soil Cd levels as input data. Eight models based on ML^17^, predicting genetic values of soybean population using artificial neural network and restricted maximum likelihood^18^, prediction of tolerant and sensitive Iranian walnut genotypes based on pomological and physicochemical traits using supervised ML models^19^ and identification of molecular markers in wheat to investigate powdery mildew, blast fungus, rust, fly larvae infection, green aphid, and Stagonospora nodorum infection^20^.

Machine learning has been previously employed for disease resistance prediction in barley^21,22^. This study distinguishes itself through several key innovations. In contrast to Hiddar et al.^21^, who focused on scald resistance prediction using genebank accessions and environmental data, and Kuska et al.^22^, who combined multispectral imaging with enzyme activity profiling for scald resistance screening, this research uniquely integrates both genotypic and phenotypic data to predict powdery mildew resistance. Furthermore, we specifically evaluate the effectiveness of various feature selection algorithms and machine learning models within this novel data integration framework. Unlike these prior studies, the dataset is derived from a field-based experiment conducted under real-world agricultural conditions in Iran, offering a distinct biological interpretation focused on identifying molecular markers and phenotypic traits relevant to powdery mildew resistance in this specific context. This work places a strong emphasis on the application of these predictive models to enhance the efficiency of barley breeding programs, a perspective that is less pronounced in previous works.

Despite the growing potential of the ML in revolutionizing plant breeding practices, there is a research gap in its application in predicting barley genotypes’ powdery mildew tolerance using molecular markers. Investigating the ML efficacy in predicting barley genotype responses to powdery mildew can lead to advancements in agricultural science. By deploying Decision Tree (DT), Random Forest (RF), Neural Network (NET), and Gaussian Process Regression (GPR) models, we aim to bridge this gap by enhancing the predictive accuracy in determining the tolerance levels of barley genotypes to powdery mildew. Through applying RReliefF, MRMR, and F-test FS algorithms, the study seeks to identify the most significant molecular markers and phenotypic traits crucial for accurately forecasting barley genotypes’ resistance to powdery mildew. The results of this study pave the way for more effective breeding programs and disease management strategies. By utilizing these methodologies, the research seeks to enhance the understanding of the genetic and phenotypic factors influencing Barley’s resistance to powdery mildew, ultimately contributing to more effective disease management strategies in agricultural practices.

## Results

**Fig. 1:**
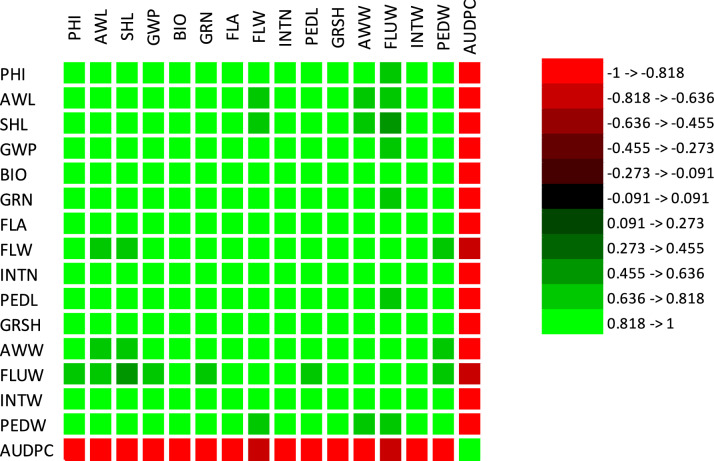
The correlation heatmap between plant height(PHI), awn length(AWL), shoot length(SHL), grain weight per spike(GWP), total biomass(BIO), number of grains(GRN), flag leaf area(FLA), flag leaf weight(FLW), Internode length(INTN), peduncle length(PEDL), grain shape(GRSH), awn weight(AWW), leaf weight under flag leaf(FLUW), Internode weight(INTW), peduncle weight(PEDW) and area under disease progress curve (AUDPC).

The traits correlations revealed that most of the correlations were negative, indicating an inverse relationship between AUDPC and the measured traits (Fig. 1). This suggested that as the values of these traits increased, the AUDPC value decreased, reflecting potentially greater resistance to powdery mildew. Strong negative correlations were observed for several key traits. For instance, awn length (AWL) exhibited a correlation coefficient of − 0.991, while internode weight (INTW) and peduncle weight (PEDW) showed coefficients of − 0.943 and − 0.993, respectively. Similarly, other traits such as plant height (PHI), shoot length (SHL), grain weight per spike (GWP), total biomass (BIO), and number of grains (GRN) demonstrated very high negative correlations, with coefficients ranging from − 0.934 to − 0.982. These results indicated that plants with larger or more robust characteristics tended to exhibit reduced disease progression, suggesting enhanced resistance to powdery mildew. Moderate negative correlations were also noted for certain traits, including flag leaf area (FLA, r =  − 0.953), awn weight (AWW, r =  − 0.851), and leaf weight under the flag leaf (FLUW, r =  − 0.709). In contrast, internode length (INTN) exhibited the weakest negative correlation (r =  − 0.657), implying it might be less directly associated with disease resistance compared to other traits. The consistent pattern of negative correlations across multiple traits suggested that these characteristics could serve as indirect indicators or predictors of powdery mildew resistance in barley breeding programs. Larger plants or those with higher biomass appeared to possess mechanisms that either reduced the severity of infection or mitigated the spread of the disease. However, it was important to note that correlation did not imply causation. The observed relationships might have been influenced by shared underlying factors, such as general plant vigor or genetic predispositions, rather than direct effects on disease resistance. From a plant disease perspective, these findings highlighted the potential for using agronomic traits as selection criteria in breeding programs aimed at improving powdery mildew resistance. Traits such as awn length, peduncle weight, and grain weight per spike emerged as particularly promising candidates due to their strong negative correlations with AUDPC.

Across our experiments, the ML models demonstrated considerable variation in their ability to predict the severity of powdery mildew in barley. This prediction was based on a combination of phenotypic traits, genotypic markers, and their interactions. The performance of each model – DT, RF, NET, and GPR – was rigorously evaluated using multiple metrics, including Mean Absolute Error (MAE), Root Mean Squared Error (RMSE), and the coefficient of determination (R2). To statistically validate the observed performance differences between the models, we further conducted Friedman’s test for pairwise comparisons across different evaluation metrics and test datasets. The results of Friedman’s test indicated statistically significant performance differences for certain model pairs, particularly when evaluated on the Phenotype and combined Phenotype-Genotype test sets, whereas fewer significant differences were observed on the Genotype test set. Furthermore, we employed three distinct feature selection algorithms, namely F-test, Minimum Redundancy Maximum Relevance (MRMR), and RReliefF, to identify the most informative attributes for the prediction task. The results, detailed in the following sections, highlight the interplay between feature selection methods and model performance, ultimately revealing the optimal strategies for accurate disease prediction.

### Attributes selected by FS algorithms

Phenotypic traits used in artificial intelligence models are shown in Table 1. These traits were selected from 15 agronomic traits. The genetic traits used in ML models are shown in Table 1. These traits were selected from 790 molecular markers.

**Table 1:** Phenotypic traits and molecular markers selected by RReliefF, MRMR, and F-test FS algorithms.

	Phenotype	Genotype
RReliefF	Grain shape, awn length, awn weight, Leaf weight under flag leaf, shoot length, plant height, peduncle weight, peduncle length, flag leaf area, Internode length	IJS1-B, HvSMEh288, ET12-29-C, iPBS2387-B, EBmac0970, iPBS2243-A, IJS12-B, Bmag0021, IJS23-A, B06-B, Bmac0273, HvSMEi872, iPBS2083-B, IJS16-B, OPB-02-B, iPBS2415-3, iPBS2391-A, Bmag0110b, OPD-07-E, B15-C
MRMR	shoot length, flag leaf area, number of grains, awn length, Leaf weight under flag leaf, plant height, grain weight per spike, Total biomass, flag leaf area, Internode length	ISSR48-4, ISSR48-1, IJS4-D, UMB310, iPBS2078-A, iPBS2273-E, EBmac0827, ET15-36-B, iPBS2220-E, OPB-04-B, IJS16-B, IJS10-A, iPBS2243-D, GBM1461, Scot4-C, scssr25538, IJS8-A, iPBS2393-B, iPBS2080-B, ET15-36-B
F-Test	shoot length, Internode length, plant height, peduncle length, peduncle weight, grain weight per spike, Grain shape, flag leaf weight, number of grains, awn weight	D03-D, IJS17-A, iPBS2415-1, IJS8-A, IJS22-A, ET15-36-B, IJS9-B, ET12-29-B, OPB-04-B, OPB-19-A, ISSR30iPBS2076-5, IJS8-E, UMB310, IJS24-A, ISSR22-1, IJS23-A, ISSR47-5, ET15-33-A, ET18-1-B, IJS4-D

The phenotypic traits and molecular markers used in this study have been previously reported as factors associated with plant morphology and genetic diversity in cereals, especially barley. However, feature selection algorithms (RReliefF, MRMR, and F-test) played a crucial role in identifying the most important subset of these predetermined traits for predicting powdery mildew resistance using machine learning models.

### Genotypic features

**Fig. 2:**
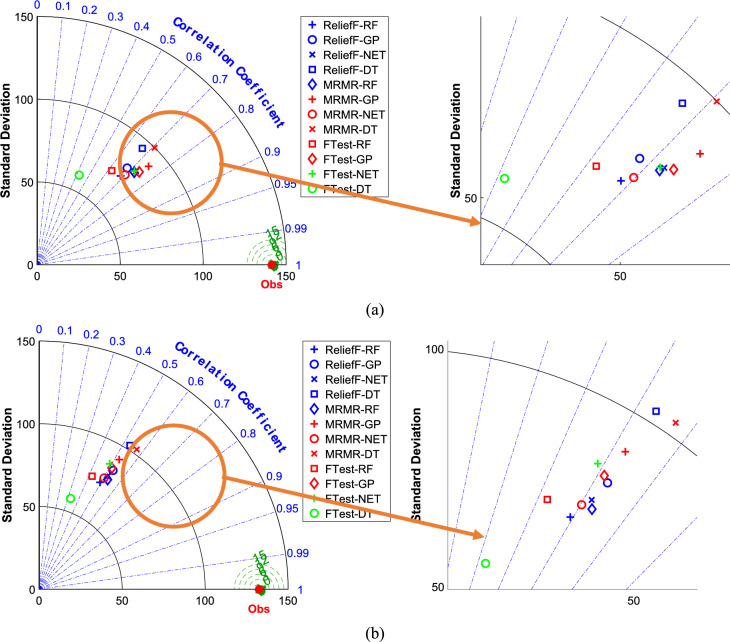
The Taylor diagrams to compare ML models predicting the severity of powdery mildew in barley lines by genotypic traits selected by F-test, MRMR, and RReliefF FS algorithms over the (**a**) train and (**b**) test datasets.

Figure 2 presents Taylor diagrams comparing the performance of the ML models (DT, RF, NET, and GPR) in predicting powdery mildew severity in barley lines using genotypic traits. We tested these models after using different feature selection methods (F-test, MRMR, and RReliefF) to pick out the most important genetic markers. Figure 2a shows the results for the training data, and Fig. 2b shows how well the models did on the data they had not seen before (the testing data).

Looking at the Taylor diagrams (Fig. 2), it is clear that the NET model outperformed other models. Its points are closest to the “Observed” marker, mainly when we used the MRMR or RReliefF feature selection methods. The RF model was not too far behind, either. Interestingly, the choice of feature selection mattered; MRMR and RReliefF consistently outperformed the F-test. We also noticed that the models did not perform quite as well on the testing data, which suggests they might have been “memorizing” the training data a bit (that is called overfitting). The DT and GPR models struggled most with this.

**Fig. 3:**
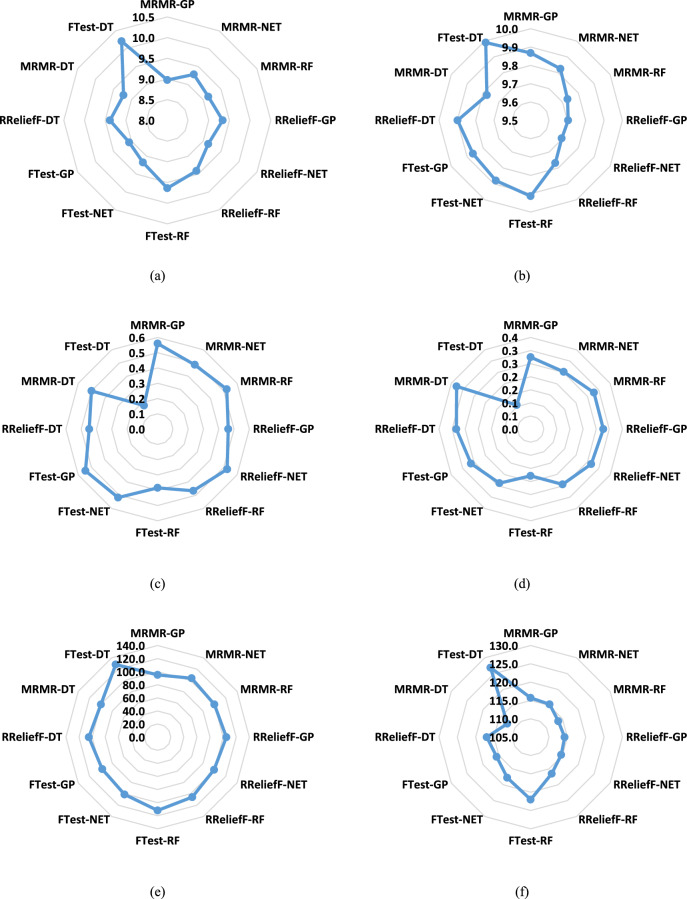
The MAE, R2, and RMSE values of ML models predicting the severity of powdery mildew in barley lines by genotypic traits selected by F-test, MRMR, and RReliefF FS algorithms over the train and test datasets: (**a**) the MAE of the training dataset, (**b**) the MAE of the test dataset, (**c**) the R^2^ of the training dataset, (**d**) the R^2^ of the test dataset, (**e**) the RMSE of the training dataset, (**f**) the RMSE of the test dataset.

Figure 3 displays the MAE, R2, RMSE values for the same ML models and feature selection methods as in Fig. 2, again using genotypic traits to predict powdery mildew severity. The training data results are in Fig. 3a, c, e, while the testing data results are in Fig. 3b, d, f. Figure 3 confirms that the NET model generally achieved the lowest MAE, RMSE, and highest R^2^ values, consistent with the Taylor diagrams, particularly on the test dataset. The RF Model shows acceptable results. The MRMR and RReliefF feature selection methods consistently outperformed the F-test across all models and metrics. A noticeable difference between training and testing set performance was observed, with higher MAE and RMSE and lower R^2^ values on the test set, reinforcing the indication of some overfitting.

### Phenotypic features

**Fig. 4:**
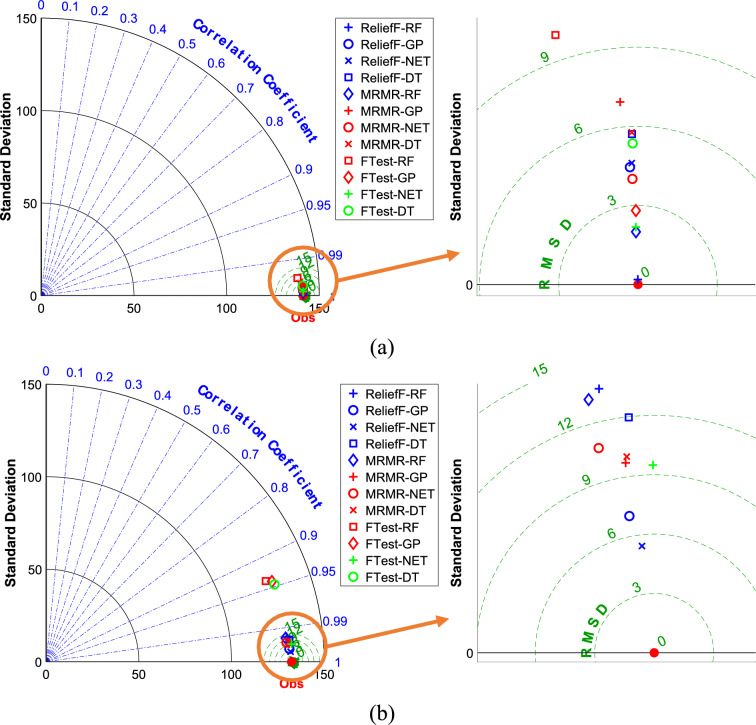
The Taylor diagrams to compare ML models predicting the severity of powdery mildew in barley lines by phenotypic traits selected by F-test, MRMR, and RReliefF FS algorithms over the (**a**) train and (**b**) test datasets.

Figure 4 shows Taylor diagrams comparing the performance of the DT, RF, NET, and GPR models in predicting powdery mildew severity in barley lines, this time using phenotypic traits. The training (Fig. 4a) and testing (Fig. 4b) datasets are presented separately. Feature selection was conducted using the F-test, MRMR, and RReliefF algorithms. The Taylor diagrams in Fig. 4 reveal that the NET model again demonstrated superior performance, with points closest to the “Observed” marker on both training and testing datasets. The RF also showed promising results. Unlike the genotypic data, the choice of feature selection method had a less pronounced impact on model performance when using phenotypic traits. However, MRMR and RReliefF still generally yielded slightly better results. The performance on the test set was comparable to, though slightly worse than, the training set. The models trained on phenotypic data may generalize somewhat better than those trained solely on genotypic data, although overfitting is still present.

**Fig. 5:**
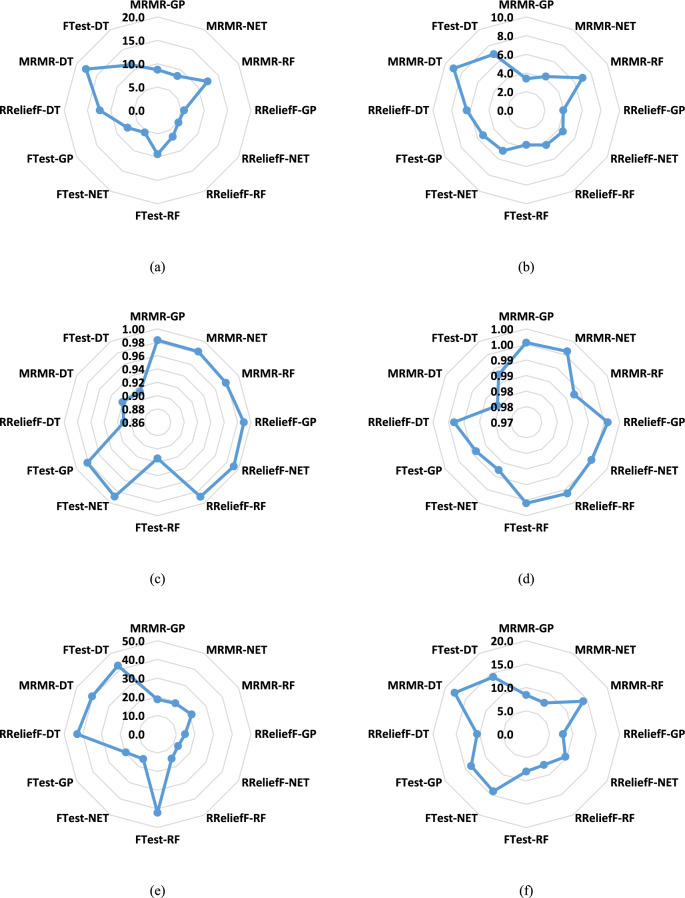
The MAE, R^2^, and RMSE values of ML models predicting the severity of powdery mildew in barley lines by phenotype traits selected by F-test, MRMR, and RReliefF FS algorithms over the train and test datasets: (**a**) the MAE of the training dataset, (**b**) the MAE of the test dataset, (**c**) the R^2^ of the training dataset, (**d**) the R^2^ of the test dataset, (**e**) the RMSE of the training dataset, (**f**) the RMSE of the test dataset.

Figure 5 presents the MAE, R^2^, and RMSE values for the same models and feature selection methods as Fig. 4, using phenotypic traits to predict powdery mildew severity. The results are shown for the training (Figs. 5a, c, e) and testing (Figs. 5b, d, f) datasets. The metrics in Fig. 5 confirm the trends observed in the Taylor diagrams. The NET model consistently exhibited the lowest MAE, RMSE, and the highest R^2^ values, indicating the best predictive performance. The RF also showed reasonable. While the differences between feature selection methods were less pronounced than with genotypic data, MRMR and RReliefF generally led to slightly improved performance compared to the F-test. The test set performance was usually worse than the training set performance, although the difference was less dramatic than observed with genotypic data. These findings further support using the NET model, potentially in combination with MRMR or RReliefF feature selection, for predicting powdery mildew resistance based on phenotypic traits.

### Phenotypic and genotypic features

**Fig. 6:**
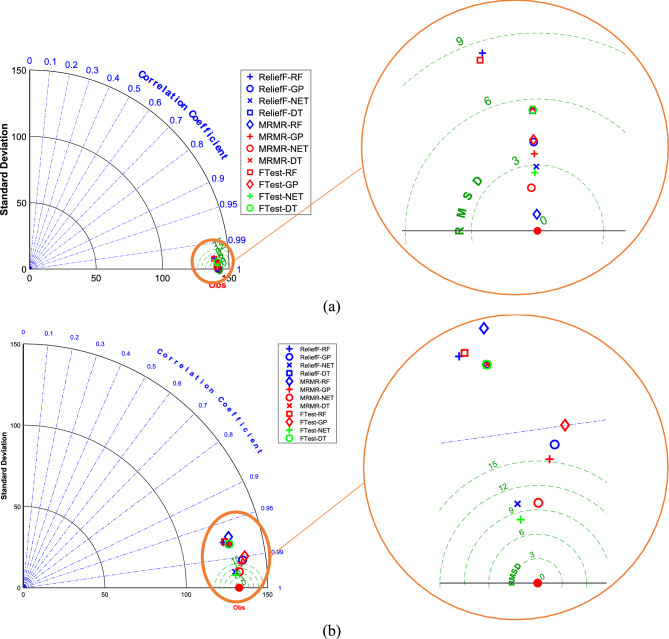
The Taylor diagrams to compare ML models predicting the severity of powdery mildew in barley lines by Phenotypic and Genotypic traits selected by F-test, MRMR, and RReliefF FS algorithms over the (**a**) train and (**b**) test datasets.

Figure 6 displays Taylor diagrams comparing the performance of the four ML models (DT, RF, NET, and GPR) in predicting powdery mildew severity, this time using a combination of both phenotypic and genotypic traits. Separate diagrams for the training (Fig. 6a) and testing (Fig. 6b) datasets are shown. The F-test, MRMR, and RReliefF algorithms were used for feature selection. The Taylor diagrams in Fig. 6 indicate that the NET model continued to exhibit the best performance, with points closest to the “Observed” marker on both the training and testing datasets. The RF model also showed good performance. The choice of feature selection method had a noticeable impact, with MRMR and RReliefF generally leading to better performance than the F-test. The test set performance was slightly worse than the training set performance, indicating some overfitting. The combined use of phenotypic and genotypic traits offers a slight improvement in predictive accuracy compared to using either trait type alone, particularly for the NET model.

**Fig. 7:**
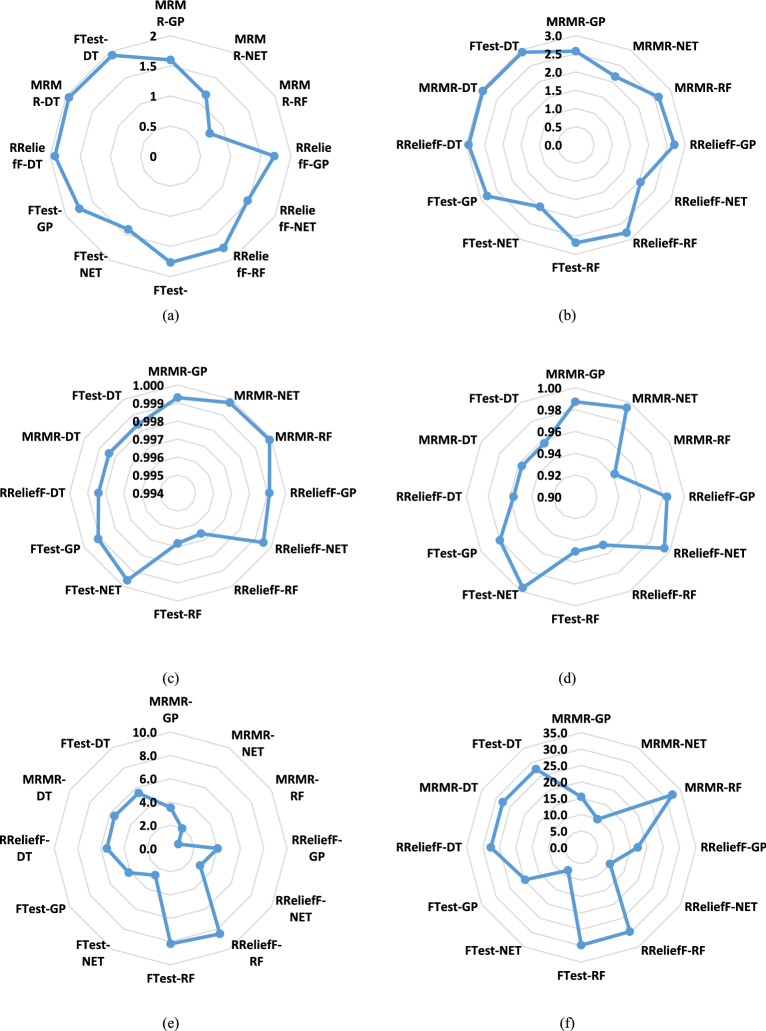
The MAE, R^2^, and RMSE values of ML models predicting the severity of powdery mildew in barley lines by phenotypic and genotypic traits selected by F-test, MRMR, and RReliefF FS algorithms over the train and test datasets: (**a**) the MAE of the training dataset, (**b**) the MAE of the test dataset, (**c**) the R^2^ of the training dataset, (**d**) the R^2^ of the test dataset, (**e**) the RMSE of the training dataset, (**f**) the RMSE of the test dataset.

Figure 7 presents the MAE, R^2^, and RMSE values for the models and feature selection methods used in Fig. 6, using the combined phenotypic and genotypic traits to predict powdery mildew severity. The results are shown separately for the training (Figs. 7a, c, e) and testing (Figs. 7b, d, f) datasets. The metrics in Fig. 7 corroborate the findings from the Taylor diagrams. The NET model consistently achieved the lowest MAE and RMSE and the highest R^2^ values, indicating superior predictive performance. The RF model also showed promising results. MRMR and RReliefF feature selection generally outperformed the F-test. The test set performance was slightly worse than the training set performance, consistent with some degree of overfitting. The results suggest that combining phenotypic and genotypic traits, along with the NET model and MRMR or RReliefF feature selection, provides the most accurate predictions of powdery mildew severity in the barley lines studied.

### Statistical tests for ML models

The following tables present the p-values from pairwise comparisons based on Friedman’s test for different evaluation metrics and test scenarios. In each table, we compare the performance of twelve models: RRF (RReliefF-RF), RGP (RReliefF-GP), RNET (RReliefF-NET), RDT (RReliefF-DT), MRF (MRMR-RF), MGP (MRMR-GP), MNET (MRMR-NET), MDT (MRMR-DT), FRF (FTest-RF), FGP (FTest-GP), FNET (FTest-NET), and FDT (FTest-DT). The rows and columns of each table represent these models, and the cell at the intersection of a row and a column contains the p-value from the pairwise comparison between the model in the row and the model in the column. For all tests, a p-value less than 0.05 is generally considered statistically significant at a 5% significance level, indicating a significant difference in performance between the two compared models for the specific metric.

In Table 2, p-values less than 0.05 indicate statistically significant differences in MAE on the phenotypic features over the test set. When comparing RDT with MNET, FNET, and FDT, the p-values are < 0.0001, < 0.0001, and < 0.0001, respectively, which are significantly below 0.05. The RDT’s performance in terms of MAE on the phenotypic features over the test set is statistically significantly different from MNET, FNET, and FDT. Conversely, comparing RRF and RGP yields a p-value of 0.998, indicating no statistically significant difference in their MAE performance on the phenotypic features over the test set. Many comparisons show p-values close to 1, resulting in no significant performance difference for MAE on the phenotypic features over the test set for those model pairs.

**Table 2:** P-values for pairwise comparisons based on the mean absolute error (MAE) metric on the phenotypic features over the test set.

	RRF	RGP	RNET	RDT	MRF	MGP	MNET	MDT	FRF	FGP	FNET	FDT
RRF	1	0.998	0.994	0.386	1.000	0.103	0.340	0.995	0.879	1.000	**0.014**	0.459
RGP	0.998	1	1.000	**0.035**	0.988	**0.004**	0.931	0.655	1.000	0.963	0.227	**0.049**
RNET	0.994	1.000	1	**0.023**	0.973	**0.003**	0.963	0.563	1.000	0.931	0.296	**0.033**
RDT	0.386	**0.035**	**0.023**	1	0.547	1.000	** < 0.0001**	0.969	**0.003**	0.680	** < 0.0001**	1.000
MRF	1.000	0.988	0.973	0.547	1	0.182	0.214	0.999	0.756	1.000	**0.006**	0.625
MGP	0.103	**0.004**	**0.003**	1.000	0.182	1	** < 0.0001**	0.714	**0.000**	0.272	** < 0.0001**	1.000
MNET	0.340	0.931	0.963	** < 0.0001**	0.214	** < 0.0001**	1	**0.021**	1.000	0.138	0.990	**0.000**
MDT	0.995	0.655	0.563	0.969	0.999	0.714	**0.021**	1	0.210	1.000	**0.000**	0.984
FRF	0.879	1.000	1.000	**0.003**	0.756	**0.000**	1.000	0.210	1	0.630	0.680	**0.005**
FGP	1.000	0.963	0.931	0.680	1.000	0.272	0.138	1.000	0.630	1	**0.003**	0.752
FNET	**0.014**	0.227	0.296	** < 0.0001**	**0.006**	** < 0.0001**	0.990	**0.000**	0.680	**0.003**	1	** < 0.0001**
FDT	0.459	**0.049**	**0.033**	1.000	0.625	1.000	**0.000**	0.984	**0.005**	0.752	** < 0.0001**	1

The compared models are RRF (RReliefF-RF), RGP (RReliefF-GP), RNET (RReliefF-NET), RDT (RReliefF-DT), MRF (MRMR-RF), MGP (MRMR-GP), MNET (MRMR-NET), MDT (MRMR-DT), FRF (FTest-RF), FGP (FTest-GP), FNET (FTest-NET), and FDT (FTest-DT).

Significant values are given in bold.

Across Table 3, most p-values are very high, often 1.000 or close to it. For the MAE metric on the genotypic features over the test set, there are generally no statistically significant differences between the performances of most model pairs. The high p-values shows that the models perform very similarly in terms of MAE when evaluated on the genotypic features over the test set, and any observed differences are likely due to random variation. All comparisons involving RRF, RGP, RNET, MRF, MGP, MNET, MDT, FRF, and FGP have p-values of 0.95 or higher when compared against each other, indicating a lack of statistically significant differences.

**Table 3:** P-values for pairwise comparisons using the MAE metric on the genotypic features over the test set.

	RRF	RGP	RNET	RDT	MRF	MGP	MNET	MDT	FRF	FGP	FNET	FDT
RRF	1	1.000	1.000	0.999	1.000	1.000	1.000	1.000	1.000	1.000	1.000	0.960
RGP	1.000	1	1.000	0.954	1.000	0.998	1.000	1.000	1.000	0.985	1.000	0.710
RNET	1.000	1.000	1	0.931	1.000	0.996	1.000	1.000	1.000	0.975	1.000	0.650
RDT	0.999	0.954	0.931	1	0.973	1.000	0.999	1.000	0.994	1.000	1.000	1.000
MRF	1.000	1.000	1.000	0.973	1	0.999	1.000	1.000	1.000	0.993	1.000	0.774
MGP	1.000	0.998	0.996	1.000	0.999	1	1.000	1.000	1.000	1.000	1.000	0.997
MNET	1.000	1.000	1.000	0.999	1.000	1.000	1	1.000	1.000	1.000	1.000	0.951
MDT	1.000	1.000	1.000	1.000	1.000	1.000	1.000	1	1.000	1.000	1.000	0.975
FRF	1.000	1.000	1.000	0.994	1.000	1.000	1.000	1.000	1	0.999	1.000	0.891
FGP	1.000	0.985	0.975	1.000	0.993	1.000	1.000	1.000	0.999	1	1.000	1.000
FNET	1.000	1.000	1.000	1.000	1.000	1.000	1.000	1.000	1.000	1.000	1	0.981
FDT	0.960	0.710	0.650	1.000	0.774	0.997	0.951	0.975	0.891	1.000	0.981	1

The compared models are RRF (RReliefF-RF), RGP (RReliefF-GP), RNET (RReliefF-NET), RDT (RReliefF-DT), MRF (MRMR-RF), MGP (MRMR-GP), MNET (MRMR-NET), MDT (MRMR-DT), FRF (FTest-RF), FGP (FTest-GP), FNET (FTest-NET), and FDT (FTest-DT).

In Table 4, we observe more statistically significant differences compared to Table 3. Comparing RDT with RRF, RNET, MRF, MNET, MDT, FRF, FNET, and FDT results in p-values of 0.001, < 0.0001, < 0.0001, < 0.0001, < 0.0001, < 0.0001, < 0.0001 and < 0.0001 respectively, all well below 0.05. The obtained p-values indicated significant differences in MAE performance on the combined Phenotypic and genotypic features over the test set. Pairs like RGP and FNET and RGP and FDT also show very low p-values (< 0.0001), indicating statistically significant differences. Conversely, comparisons like RRF and MRF (p = 1.000) and RRF and MNET (p = 0.997) indicate no significant differences.

**Table 4:** P-values for pairwise comparisons based on the MAE metric on the combined phenotypic and genotypic features over the test set.

	RRF	RGP	RNET	RDT	MRF	MGP	MNET	MDT	FRF	FGP	FNET	FDT
RRF	1	0.235	0.738	**0.001**	1.000	0.747	0.997	**0.002**	1.000	0.415	0.485	**0.002**
RGP	0.235	1	**0.000**	0.899	**0.046**	1.000	**0.013**	0.913	**0.029**	1.000	** < 0.0001**	0.910
RNET	0.738	**0.000**	1	** < 0.0001**	0.979	**0.008**	0.999	** < 0.0001**	0.992	**0.001**	1.000	** < 0.0001**
RDT	**0.001**	0.899	** < 0.0001**	1	** < 0.0001**	0.410	** < 0.0001**	1.000	** < 0.0001**	0.743	** < 0.0001**	1.000
MRF	1.000	**0.046**	0.979	** < 0.0001**	1	0.313	1.000	**0.000**	1.000	0.107	0.885	** < 0.0001**
MGP	0.747	1.000	**0.008**	0.410	0.313	1	0.133	0.434	0.235	1.000	**0.002**	0.429
MNET	0.997	**0.013**	0.999	** < 0.0001**	1.000	0.133	1	** < 0.0001**	1.000	**0.035**	0.982	** < 0.0001**
MDT	**0.002**	0.913	** < 0.0001**	1.000	**0.000**	0.434	** < 0.0001**	1	** < 0.0001**	0.765	** < 0.0001**	1.000
FRF	1.000	**0.029**	0.992	** < 0.0001**	1.000	0.235	1.000	** < 0.0001**	1	0.072	0.936	** < 0.0001**
FGP	0.415	1.000	**0.001**	0.743	0.107	1.000	**0.035**	0.765	0.072	1	**0.000**	0.761
FNET	0.485	** < 0.0001**	1.000	** < 0.0001**	0.885	**0.002**	0.982	** < 0.0001**	0.936	**0.000**	1	** < 0.0001**
FDT	**0.002**	0.910	** < 0.0001**	1.000	** < 0.0001**	0.429	** < 0.0001**	1.000	** < 0.0001**	0.761	** < 0.0001**	1

The compared models are RRF (RReliefF-RF), RGP (RReliefF-GP), RNET (RReliefF-NET), RDT (RReliefF-DT), MRF (MRMR-RF), MGP (MRMR-GP), MNET (MRMR-NET), MDT (MRMR-DT), FRF (FTest-RF), FGP (FTest-GP), FNET (FTest-NET), and FDT (FTest-DT).

Significant values are given in bold.

Similar to Table 2, Table 5 for RMSE on the phenotypic features over the test set shows several statistically significant differences. Comparisons involving RDT with MNET and FNET yield p-values of < 0.0001, highlighting significant performance differences in terms of RMSE on the phenotypic features over the test set. The interpretations are analogous to Table 2 but based on the RMSE metric instead of MAE.

**Table 5:** P-values for pairwise comparisons based on the RMSE metric on the phenotypic features over the test set.

	RRF	RGP	RNET	RDT	MRF	MGP	MNET	MDT	FRF	FGP	FNET	FDT
RRF	1	0.998	0.994	0.386	1.000	0.103	0.340	0.995	0.879	1.000	**0.014**	0.459
RGP	0.998	1	1.000	**0.035**	0.988	**0.004**	0.931	0.655	1.000	0.963	0.227	**0.049**
RNET	0.994	1.000	1	**0.023**	0.973	**0.003**	0.963	0.563	1.000	0.931	0.296	**0.033**
RDT	0.386	**0.035**	**0.023**	1	0.547	1.000	** < 0.0001**	0.969	**0.003**	0.680	** < 0.0001**	1.000
MRF	1.000	0.988	0.973	0.547	1	0.182	0.214	0.999	0.756	1.000	**0.006**	0.625
MGP	0.103	**0.004**	**0.003**	1.000	0.182	1	** < 0.0001**	0.714	**0.000**	0.272	** < 0.0001**	1.000
MNET	0.340	0.931	0.963	** < 0.0001**	0.214	** < 0.0001**	1	**0.021**	1.000	0.138	0.990	**0.000**
MDT	0.995	0.655	0.563	0.969	0.999	0.714	**0.021**	1	0.210	1.000	**0.000**	0.984
FRF	0.879	1.000	1.000	**0.003**	0.756	**0.000**	1.000	0.210	1	0.630	0.680	**0.005**
FGP	1.000	0.963	0.931	0.680	1.000	0.272	0.138	1.000	0.630	1	**0.003**	0.752
FNET	**0.014**	0.227	0.296	** < 0.0001**	**0.006**	** < 0.0001**	0.990	**0.000**	0.680	**0.003**	1	** < 0.0001**
FDT	0.459	**0.049**	**0.033**	1.000	0.625	1.000	**0.000**	0.984	**0.005**	0.752	** < 0.0001**	1

The compared models are RRF (RReliefF-RF), RGP (RReliefF-GP), RNET (RReliefF-NET), RDT (RReliefF-DT), MRF (MRMR-RF), MGP (MRMR-GP), MNET (MRMR-NET), MDT (MRMR-DT), FRF (FTest-RF), FGP (FTest-GP), FNET (FTest-NET), and FDT (FTest-DT).

Significant values are given in bold.

Consistent with results from Table 3, Table 6 also largely shows high p-values close to 1.000. For the RMSE metric on the genotypic features over the test set, there are generally no statistically significant differences between the performances of most model pairs. The models exhibit similar RMSE performance when evaluated on the genotypic features over the test set.

**Table 6:** P-values for pairwise comparisons using the RMSE metric on the genotypic features over the test set.

	RRF	RGP	RNET	RDT	MRF	MGP	MNET	MDT	FRF	FGP	FNET	FDT
RRF	1	1.000	1.000	0.999	1.000	1.000	1.000	1.000	1.000	1.000	1.000	0.960
RGP	1.000	1	1.000	0.954	1.000	0.998	1.000	1.000	1.000	0.985	1.000	0.710
RNET	1.000	1.000	1	0.931	1.000	0.996	1.000	1.000	1.000	0.975	1.000	0.650
RDT	0.999	0.954	0.931	1	0.973	1.000	0.999	1.000	0.994	1.000	1.000	1.000
MRF	1.000	1.000	1.000	0.973	1	0.999	1.000	1.000	1.000	0.993	1.000	0.774
MGP	1.000	0.998	0.996	1.000	0.999	1	1.000	1.000	1.000	1.000	1.000	0.997
MNET	1.000	1.000	1.000	0.999	1.000	1.000	1	1.000	1.000	1.000	1.000	0.951
MDT	1.000	1.000	1.000	1.000	1.000	1.000	1.000	1	1.000	1.000	1.000	0.975
FRF	1.000	1.000	1.000	0.994	1.000	1.000	1.000	1.000	1	0.999	1.000	0.891
FGP	1.000	0.985	0.975	1.000	0.993	1.000	1.000	1.000	0.999	1	1.000	1.000
FNET	1.000	1.000	1.000	1.000	1.000	1.000	1.000	1.000	1.000	1.000	1	0.981
FDT	0.960	0.710	0.650	1.000	0.774	0.997	0.951	0.975	0.891	1.000	0.981	1

The compared models are RRF (RReliefF-RF), RGP (RReliefF-GP), RNET (RReliefF-NET), RDT (RReliefF-DT), MRF (MRMR-RF), MGP (MRMR-GP), MNET (MRMR-NET), MDT (MRMR-DT), FRF (FTest-RF), FGP (FTest-GP), FNET (FTest-NET), and FDT (FTest-DT).

Similar to Table 4, Table 7 reveals statistically significant differences in RMSE performance for certain model pairs on the combined Phenotypic and genotypic features over the test set. Comparisons of RDT with models such as MNET, FNET, and FDT result in p-values less than 0.0001, signifying statistically significant differences. These results parallel the findings from Table 4 but are based on the RMSE metric, reinforcing the observed performance distinctions on the combined dataset.

**Table 7:** P-values for pairwise comparisons based on the RMSE metric on the combined phenotypic and genotypic features over the test set.

	RRF	RGP	RNET	RDT	MRF	MGP	MNET	MDT	FRF	FGP	FNET	FDT
RRF	1	0.235	0.738	**0.001**	1.000	0.747	0.997	**0.002**	1.000	0.415	0.485	**0.002**
RGP	0.235	1	**0.000**	0.899	**0.046**	1.000	**0.013**	0.913	**0.029**	1.000	** < 0.0001**	0.910
RNET	0.738	**0.000**	1	** < 0.0001**	0.979	**0.008**	0.999	** < 0.0001**	0.992	**0.001**	1.000	** < 0.0001**
RDT	**0.001**	0.899	** < 0.0001**	1	** < 0.0001**	0.410	** < 0.0001**	1.000	** < 0.0001**	0.743	** < 0.0001**	1.000
MRF	1.000	**0.046**	0.979	** < 0.0001**	1	0.313	1.000	**0.000**	1.000	0.107	0.885	** < 0.0001**
MGP	0.747	1.000	**0.008**	0.410	0.313	1	0.133	0.434	0.235	1.000	**0.002**	0.429
MNET	0.997	**0.013**	0.999	** < 0.0001**	1.000	0.133	1	** < 0.0001**	1.000	**0.035**	0.982	** < 0.0001**
MDT	**0.002**	0.913	** < 0.0001**	1.000	**0.000**	0.434	** < 0.0001**	1	** < 0.0001**	0.765	** < 0.0001**	1.000
FRF	1.000	**0.029**	0.992	** < 0.0001**	1.000	0.235	1.000	** < 0.0001**	1	0.072	0.936	** < 0.0001**
FGP	0.415	1.000	**0.001**	0.743	0.107	1.000	**0.035**	0.765	0.072	1	**0.000**	0.761
FNET	0.485	** < 0.0001**	1.000	** < 0.0001**	0.885	**0.002**	0.982	** < 0.0001**	0.936	**0.000**	1	** < 0.0001**
FDT	**0.002**	0.910	** < 0.0001**	1.000	** < 0.0001**	0.429	** < 0.0001**	1.000	** < 0.0001**	0.761	** < 0.0001**	1

The compared models are RRF (RReliefF-RF), RGP (RReliefF-GP), RNET (RReliefF-NET), RDT (RReliefF-DT), MRF (MRMR-RF), MGP (MRMR-GP), MNET (MRMR-NET), MDT (MRMR-DT), FRF (FTest-RF), FGP (FTest-GP), FNET (FTest-NET), and FDT (FTest-DT).

Significant values are given in bold.

### Regression between actual disease severity values and predicted values for the training dataset

**Fig. 8:**
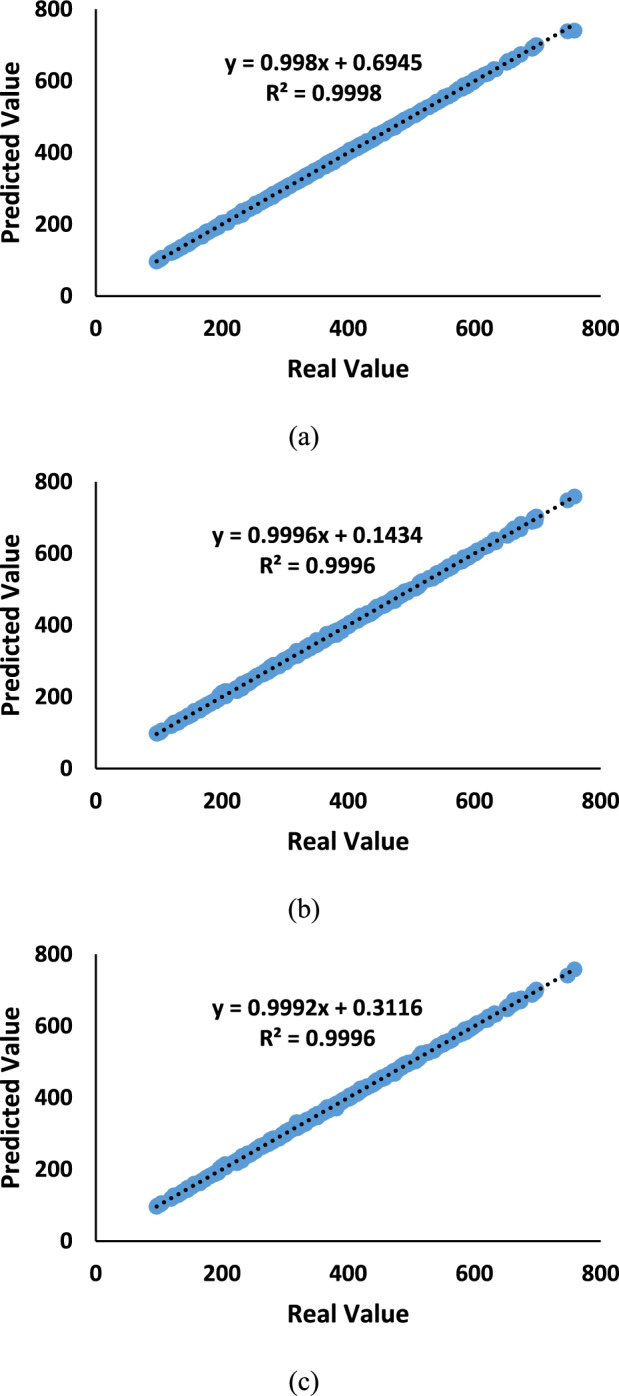
The results of the regression between actual disease severity values and predicted values by the NET model over the training dataset, focusing on the (**a**) RReliefF, (**b**) MRMR, and (**c**) F-Test FS algorithms.

The results of the regression between actual disease severity values and predicted values by the NET model over the training dataset, focusing on the feature selection algorithms F-Test, MRMR, and RReliefF, are indicated in Fig. 8.

The RReliefF algorithm showcases coefficient values of 0.998 for ‘a’ and 0.6945 for ‘b,’ resulting in an exceptional R^2^ value of 0.9998. The high R^2^ value suggests that the RReliefF algorithm has excelled in selecting relevant features highly correlated with the actual disease severity values. The relatively higher ‘b’ coefficient compared to the F-Test and MRMR algorithms indicates that the features chosen by RReliefF have a more substantial impact on predicting powdery mildew resistance. The results highlight the feature selection algorithms’ effectiveness in enhancing the NET model’s predictive capabilities for assessing powdery mildew resistance in barley lines using molecular markers and ML techniques (Fig. 8a).

The MRMR feature selection algorithm displays coefficient values of 0.9996 for ‘a’ and 0.1434 for ‘b,’ resulting in an R^2^ value of 0.9996. The high R^2^ value implies that the MRMR algorithm has also effectively identified important features that contribute significantly to predicting disease severity in Barley. Despite having a lower ‘b’ coefficient than the F-Test algorithm, the MRMR algorithm demonstrates predictive solid power, indicating that the selected features play a crucial role in determining the resistance of barley lines to powdery mildew (Fig. 8b).

The regression results show that the F-Test feature selection algorithm yields coefficient values of 0.9992 for ‘a’ and 0.3116 for ‘b,’ resulting in an impressive R^2^ value of 0.9996. The high R^2^ value indicates that the F-Test algorithm has successfully captured the relationship between the actual disease severity values and the predicted values generated by the NET model. The features selected by the F-Test algorithm are highly relevant for predicting powdery mildew resistance in barley lines, leading to accurate and reliable predictions (Fig. 8c).

### Regression between actual disease severity values and predicted values for the test dataset

**Fig. 9:**
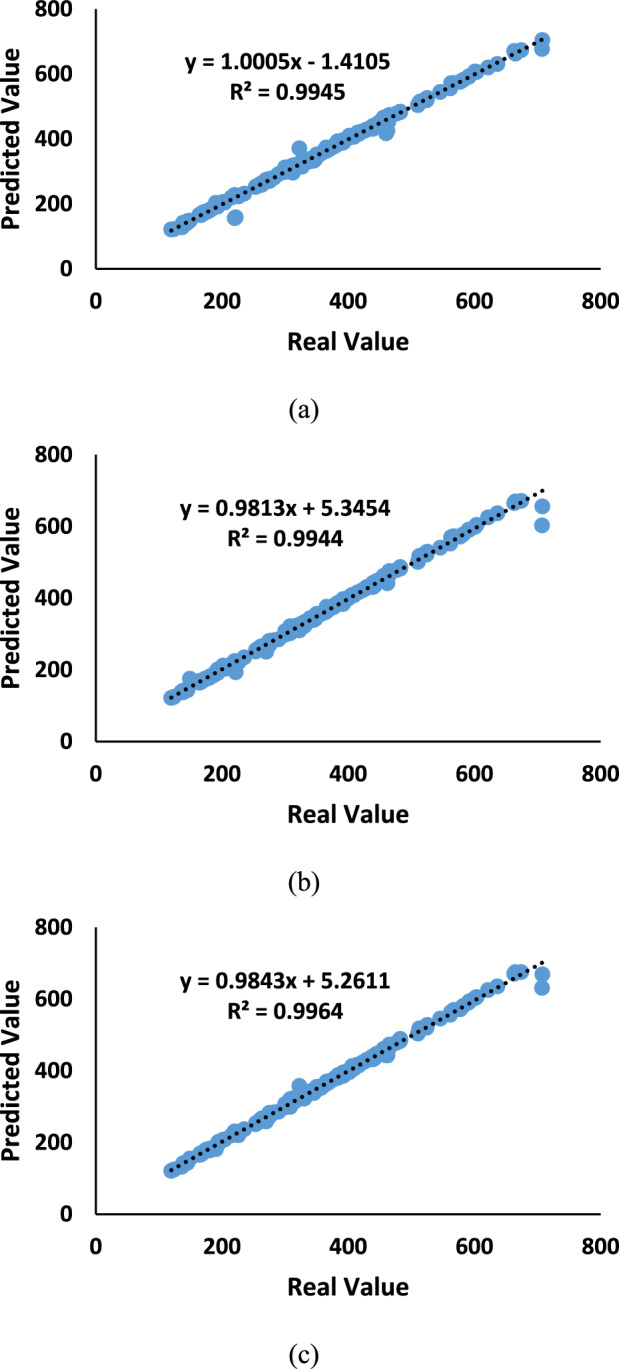
The results of the regression between actual disease severity values and predicted values by the NET model over the test dataset, focusing on the (**a**) RReliefF, (**b**) MRMR, and (**c**) F-test FS algorithms.

The regression results between the actual disease severity values and the predicted values by the NET model over the test dataset are presented in Fig. 9. The RReliefF feature selection algorithm shows coefficients ‘a’ and ‘b’ of 1.0005 and − 1.4105, respectively, with an R^2^ value of 0.9945. The high R^2^ value suggests that this algorithm also provides a reliable estimation of the disease severity based on the molecular markers and ML models used in the study. (Fig. 9a). Regarding the MRMR feature selection algorithm, the regression coefficients ‘a’ and ‘b’ are 0.9813 and 5.3454, respectively, with an R^2^ value of 0.9944. Although slightly lower than the F-Test results, the MRMR algorithm still demonstrates a robust predictive capability in estimating disease severity values. (Fig. 9b).

For the F-Test feature selection algorithm, the regression coefficients ‘a’ and ‘b’ are 0.9843 and 5.2611, respectively, resulting in a high R^2^ of 0.9964. The F-Test algorithm has effectively captured the relationship between the actual disease severity values and the predicted values by the NET model. The high R^2^ value suggests that the model explains 99.64% of the variance in the data, showcasing a robust performance (Fig. 9c).

## Discussion

The results of this study underscore the remarkable potential of the ML models, particularly the NET model, in predicting powdery mildew resistance in barley. What’s truly exciting is how well the NET model, especially when combined with the MRMR or RReliefF feature selection methods, could pinpoint the key genetic markers and observable traits linked to disease resistance. The NET consistently outperformed other models like DT and RF, achieving significantly lower prediction errors and higher correlations with actual disease severity. The NET’s ability to capture complex, non-linear relationships within the data is crucial for accurately modeling the intricate interplay of factors that determine a plant’s resistance.

One aspect that deserves particular attention is the role of feature selection. The choice of feature selection algorithm – F-test, MRMR, or RReliefF – had a tangible impact on the predictive accuracy. While all three methods contributed to reducing data dimensionality, MRMR and RReliefF generally led to better results, especially when dealing with genotypic data. This makes sense because these algorithms are designed to not only identify relevant features but also to minimize redundancy among them. In other words, they help to select a concise set of informative markers that, together, provide a strong signal for predicting disease resistance. It is also worth pointing out that while the models performed admirably on the training data, there was a noticeable, albeit small, drop in performance on the unseen test data. This phenomenon, known as overfitting, is a common challenge in ML. Future research could explore techniques like cross-validation with larger datasets, or regularization methods within the NET architecture, to further mitigate overfitting and improve the models’ ability to make accurate predictions on entirely new barley lines. The slight improvement observed when combining both phenotypic and genotypic data suggests that a holistic approach, integrating multiple layers of information, is the most promising path forward.

It is crucial to address the observation that, whereas the models exhibited strong performance on the training data, a slight decrease in performance was noted on the unseen test data. This phenomenon, known as overfitting, is a common challenge in machine learning. Overfitting occurs when a model learns the training data too well, capturing noise and specific patterns that do not generalize to new, unseen data.

To mitigate overfitting, we employed five-fold cross-validation during the training phase, as detailed in the “Materials and methods” section. This technique provides a more robust estimate of model performance on unseen data by partitioning the data into five subsets, training the model on four subsets, and evaluating it on the remaining subset, repeating this process five times. The observed performance difference between training and testing sets reveals that some degree of overfitting may still be present. To further address this issue and enhance the generalizability of the models, several strategies will be explored in future research. These combined strategies are expected to significantly improve the generalizability and robustness of the models for predicting powdery mildew resistance in unseen barley lines.

*Dataset Expansion:* Increasing the size of the dataset is a fundamental approach to combat overfitting. A larger and more diverse dataset would provide the models with a broader range of examples, reducing the likelihood of learning spurious correlations specific to the current dataset. We are actively exploring opportunities to expand the dataset through additional experiments and collaborations.

*Regularization Techniques:* Incorporating regularization techniques into the NET model architecture is a powerful method to prevent overfitting. We will investigate the following regularization methods. L1 Regularization adds a penalty term to the loss function proportional to the absolute value of the model’s weights. This process encourages sparsity, effectively forcing some weights to become zero, leading to a simpler model. L2 Regularization adds a penalty term proportional to the square of the model’s weights. The regularization encourages smaller weights, reducing the model’s complexity and preventing it from relying too heavily on any single feature. Dropout randomly deactivates a fraction of neurons during each training iteration. The network forced to learn more robust and redundant representations, preventing it from becoming overly reliant on specific neurons.

*Hyperparameter Optimization:* The Bayesian optimization was used to tune the hyperparameters, but a more extensive search, potentially exploring different optimization algorithms and wider ranges of hyperparameter values, could lead to model configurations that are less prone to overfitting.

We acknowledge that the current study is limited to the evaluation of a specific population of barley lines derived from a cross between the ‘Badia’ and ‘Kavir’ cultivars. However, it is important to emphasize that data were collected from field experiments, representing real-world agricultural conditions and providing valuable insights into disease resistance under such environments. A critical step in validating the predictive power and broader applicability of these machine learning models is to assess their performance on a more diverse range of barley genotypes and under varying environmental conditions. Ideally, future research should involve testing the trained models on independent datasets collected from different geographic locations, multiple growing seasons, varying disease pressures and diverse genetic backgrounds. Such an expanded evaluation would provide a more comprehensive and robust assessment of the models’ generalizability and their potential for application in diverse barley breeding programs worldwide. We are actively seeking collaborations and opportunities to access such datasets to conduct these broader validation studies in future research.

Furthermore, it is important to consider the broader applicability of the presented approach in comparison to studies employing more specialized techniques for disease resistance assessment, such as multispectral imaging, enzyme activity profiling, or gene expression analysis. These methods offer valuable detailed insights and their implementation can be resource-intensive and may not be readily scalable for routine high-throughput screening in diverse breeding programs. The machine learning framework, leveraging readily available phenotypic and genotypic data, presents a more pragmatic and potentially more generalizable strategy for predicting disease resistance across diverse barley genotypes and potentially in other crop species. The focus on accessible data types and robust machine learning techniques enhances the translational potential of the methodology for wider application in plant breeding and agricultural research.

This study utilized previously reported phenotypic traits and molecular markers associated with barley resistance to powdery mildew, its innovation lies in the application of machine learning methods to predict disease resistance and, more importantly, in identifying the most relevant features for accurate prediction using feature selection algorithms. The findings reinforce the importance of these known traits and markers in powdery mildew resistance, but significantly expand current understanding by demonstrating the effectiveness of machine learning models, particularly neural networks, in utilizing these features for accurate and efficient disease prediction. The use of feature selection algorithms further refines this approach by highlighting the most informative subsets of phenotypic and genotypic traits, enhancing the practical applicability of these models in breeding programs. Therefore, this study presents a methodological advancement in employing machine learning for disease resistance prediction, providing breeders with a powerful tool to prioritize and utilize existing knowledge for crop improvement.

## Conclusion

Through marker analysis, breeders can judiciously determine the plants suitable for further breeding. Leveraging AI methodologies such as ML and data analytics with molecular marker data can expedite breeding procedures. AI algorithms can discern patterns and correlations within extensive datasets, aiding breeders in efficiently pinpointing markers linked to desired characteristics. This approach empowers breeders to make informed selections for crossbreeding and subsequent breeding stages, resulting in resource and time savings. Furthermore, AI can predict the performance of nascent plant varieties based on molecular marker insights. By training models on historical data, AI algorithms can forecast the potential yield, disease resistance, and other traits of a new variety even before cultivation. This predictive capacity enables breeders to prioritize promising candidates and refine their breeding strategies. Ultimately, the amalgamation of molecular markers and artificial intelligence in plant breeding facilitates a more precise and effective identification of desirable traits, culminating in advancing plant varieties with superior attributes.

Integrating molecular markers and ML models presents a promising avenue to revolutionize the identification of genetic resistance to powdery mildew in Barley. While molecular markers offer valuable insights into genetic variations and trait control, their inability to directly target specific genes necessitates the exploration of advanced methodologies. The utilization of feature selection algorithms and ML models in this study offers a systematic approach to discerning the critical indicators of barley genotype tolerance to powdery mildew. By applying ReliefF, MRMR, and F-test algorithms, researchers can identify the most influential features contributing to barley genotypes’ resistance against powdery mildew diseases. Subsequent comparison of ML models such as DT, RF, NET, and GPR enables a comprehensive evaluation of their predictive capabilities in determining the tolerance levels of barley genotypes. This research methodology enhances the precision of predicting barley genotype tolerance. It affords valuable insights into the genetic and phenotypic factors that underpin resistance mechanisms, thereby facilitating the improvement of targeted strategies for disease management in barley cultivation.

We acknowledge that the current study is limited to assessing a specific population. An important future step would be to validate the predictive power and evaluate the performance of the models on a more diverse range of barley genotypes and under different environmental conditions. Such a broader evaluation could provide a more comprehensive and robust assessment of the generalizability of the models.

## Materials and methods

### Plant materials, experiment condition, and measurement of phenotypic data

**Fig. 10:**
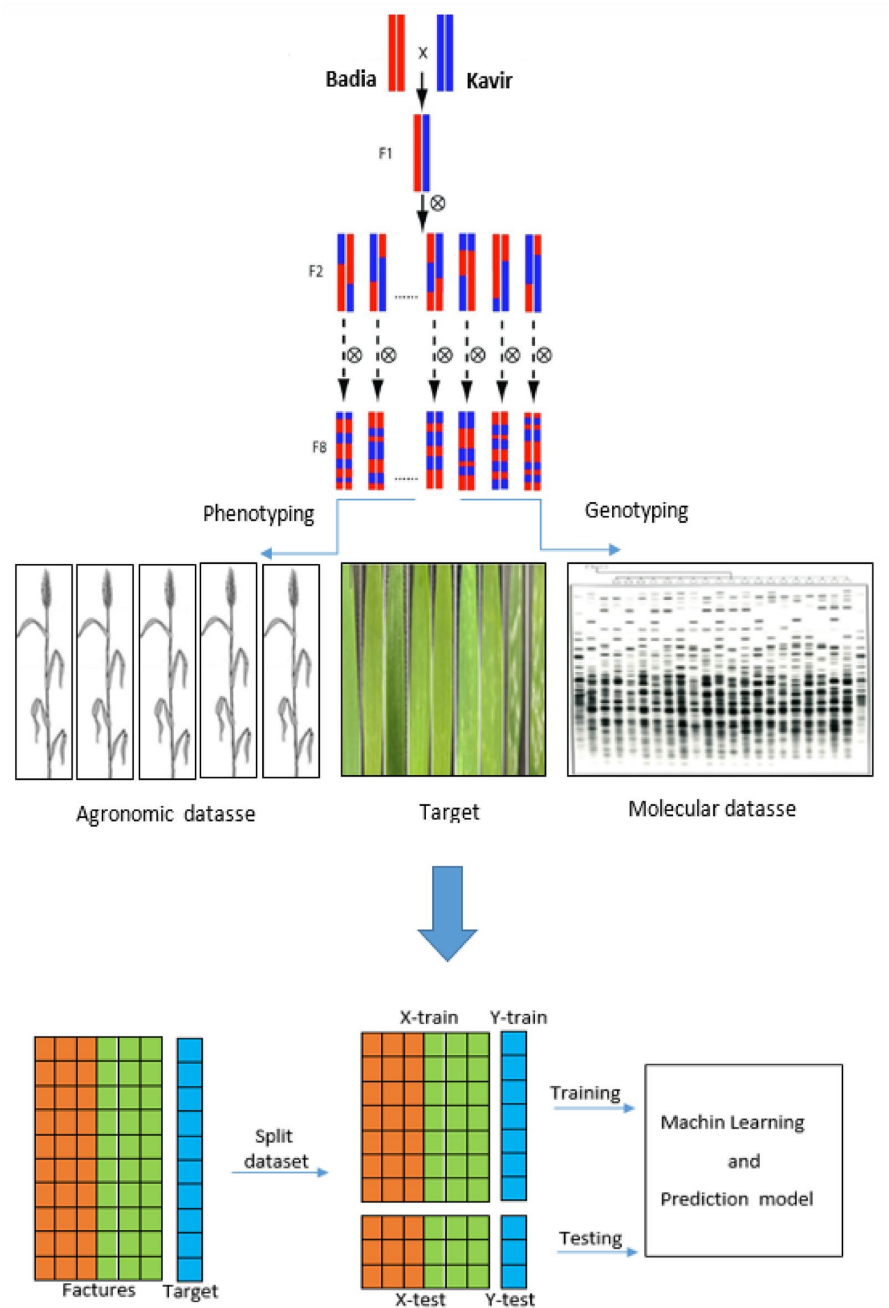
The road map of Powdery mildew resistance prediction in Barley (*Hordeum* Vulgare) based on agronomic and molecular data using a ML algorithm.

The road map of this project is shown schematically in Fig. 10. A population of barley plants was created by crossing two cultivars, Badia (female) and Kavir (male). This population was then used to develop 103 F8-F9 lines using the SSD method. The varieties Badia (head heat sensitive) and Kavir (terminal heat tolerant) were licensed under ICARDA (International Center for Agricultural Research in Dry Areas) and SPII (Seed and Plant Improvement Institute). The F8-F9 lines were grown at the Gonbad Kavous University Research Farm in 2018/2019 and 2019/2020 using an α-lactic model on three planting dates (19 November, 19 January and 19 March) at three replicates.

The F8-F9 lines were grown at the Gonbad Kavous University Research Farm in 2018/2019 and 2019/2020 using an α-lactic model on three planting dates (19 November, 19 January and 19 March) at three replicates. Planting dates were set so the genotypes were exposed to various temperature and rainfall changes during planting and ripening. In this way, multiple data was obtained, and this issue became suitable for estimation and prediction. Compared to datasets in previous powdery mildew resistance studies, the dataset used in this research offers significant improvements in terms of size, genetic diversity, and environmental variations. The dataset is composed of a relatively large F8-F9 population of 130 barley lines, derived from a cross between genetically diverse cultivars (‘Badia’ and ‘Kavir’), thus encompassing considerable genetic diversity relevant to powdery mildew resistance. Furthermore, experiments were conducted across three distinct planting dates in two consecutive years (2018/2019 and 2019/2020), explicitly incorporating environmental variations. This multi-environment approach ensures the dataset is well-suited for analyzing genotype-by-environment interactions and developing robust prediction models applicable across diverse environmental conditions. In this research agronomically features were recorded. Theses features were containing plant height(PHI), awn length(AWL), shoot length(SHL), grain weight per spike(GWP), total biomass(BIO), number of grains(GRN), flag leaf area(FLA), flag leaf weight(FLW), Internode length(INTN), peduncle length(PEDL), grain shape(GRSH), awn weight(AWW), leaf weight under flag leaf(FLUW), Internode weight(INTW) and peduncle weight(PEDW).

Planting dates were set so the genotypes were exposed to various temperature and rainfall changes during planting and ripening. In this way, multiple data was obtained, and this issue became suitable for estimation and prediction.

**Fig. 11:**
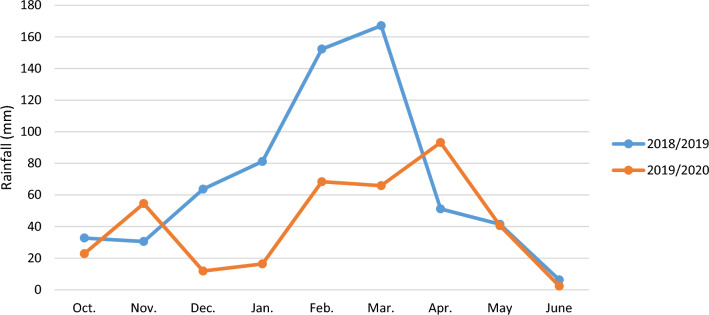
Rainfall of the experimental site in the Gonbad Kavous region during 2018/2019 and 2019/2020.

**Fig. 12:**
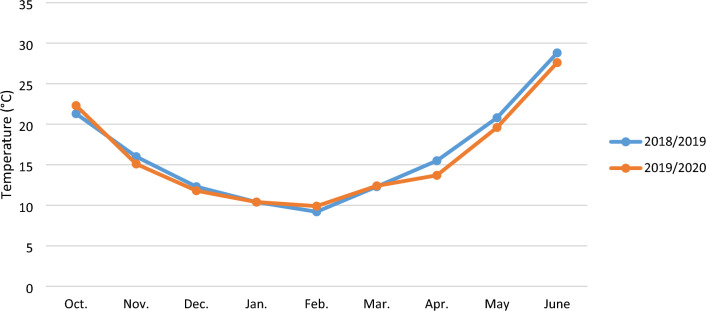
The mean temperature of the experimental site in the Gonbad Kavous region during the 2018/2019 and 2019/2020.

In this study, the late sowing was determined based on the general meteorological data of the Gonbad Kavous region (Figs. 11, 12). Each line was planted in two rows, with a row spacing of 20 cm and a 270 seeds/m^2^ plant density. Nutrient requirements were determined based on a soil test (Table 8). All crop management was done according to international practices and methods.

**Table 8:** Physical and chemical properties of used soil.

Cu (ppm)	Zn (ppm)	Mn (ppm)	Fe (ppm)	Lime (%)	pH	EC (ds/m)	SP^b^	OC^a^ (%)	Sand (%)	Silt (%)	Clay (%)	K (ppm)	P (ppm)	N (%)	Soil texture
1.6	0.6	16	2.8	10.8	7.6	0.96	48.5	0.78	13	36	31	340	13	0.08	Silt–clay-loam

^a^SP: Saturation percentage.

^b^OC: Organic compound.

**Fig. 13:**
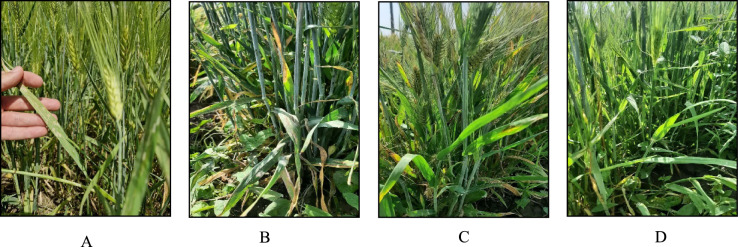
View of healthy and powdery mildew infected plants. From (**A**–**D**), the severity of the disease has decreased.

The susceptible cultivar ‘Afzal’ was planted between and around the outside of the field trial as disease spreader. Fertilizing and other crop care followed international protocols. Infected Barley leaves by *B. graminis* fsp. *tritici*-Gonbad isolates were collected during the respective trial seasons from barley fields at the Golestan Agricultural and Natural Resources Research and Education Center (AREEO). In greenhouse (20 ± 2 °C, 70–90% relative humidity under 16 h photoperiod), leaves of one-week-old seedlings of susceptible cultivar, Afzal, were subjected to inoculation by shaking the conidia of infected leaves over the healthy leaves of Afzal seedlings. After 8–10 days, various pots of susceptible cultivars with fully developed pathogen colonies were placed uniformly between lines in the field. Inoculation was performed in the fourth stage of Zadoks decimal code at the booting stage (Fig. 13).

The disease evaluation was conducted weekly after the first occurrence of disease symptoms in the susceptible cultivar using a double-digit scale (00–99)^23^. Area Under Disease Progress Curve (AUDPC) was calculated as^24^:

1 $$\text{AUDPC}={\sum }_{i=1}^{n}\frac{({x}_{i+1}+{x}_{i})({t}_{i+1}-{t}_{i})}{2}$$

where x_i_: disease severity at t_i_, xi + 1: disease severity at t_i+1_, t_i+1_-t_i_: time intervals (days) between two records of the disease, n: number of recording times.

### The measurement of genotypic data

To isolate genomic DNA, 3–4 fresh leaf samples were taken from random plants, and then the genomic DNA of the leaf samples was extracted using the CTAB method^25^. Spectrophotometry and horizontal agarose gel electrophoresis on a 0.8% agarose gel were used to ensure sufficient quantity and quality of genomic DNA. The SSR markers were used as anchors in constructing a genetic map^26^. In addition, Inter Simple Sequence Repeat (ISSR)^27^, Random Amplified Polymorphic DNA (RAPD)^28^, interprimer binding site (iPBS)^29^, inter retrotransposon amplified polymorphism (IRAP)^30^, Start Codon Target (SCoT), CAAT Box-Derived Polymorphism (CBDP)^31^, ISSR-iPBS combined and iPBS- iPBS combined were used. SSR markers were selected to have at least one marker on each chromosome arm (http://wheat.pw.usda.gov/GG3/). 10 µL of polymerase chain reaction (PCR) reaction mixture for SSR markers contained 2.5 µL DNA, 0.48 µL MgCl_2_ (50 mM), 0.6 µL dNTP (10 mM), 0.75 µL pmol primer (10 µl primer), 0.75 µl revision primer (10 pmol), 1 µl PCR buffer, 0.12 µl Taq DNA polymerase enzyme (5 U/ml) and 3.8 µl sterile distilled water. The PCR reaction mix for the dominant markers was identical, except that 1.5 µl primers were used. PCR was performed using a Thermal Cycler (Bio-Rad, USA) under the contact heat program^32^. Vertical polyacrylamide gel electrophoresis on a 6% polyacrylamide gel was used to separate the layers, and gel staining was performed using the silver nitrate method^33^. Finally 719 PolyMorphic bands were used as genotypic features.

### Data preprocessing

Prior to the application of feature selection algorithms and machine learning models, the dataset underwent rigorous preprocessing and quality control procedures to ensure data integrity and consistency. These steps are Data Cleaning, Missing Value Handling, Data Transformation and Data Type Consistency.

*Data cleaning:* A comprehensive inspection of both phenotypic and genotypic data was performed to identify and rectify any inconsistencies, errors, or outliers. Erroneous data points, such as negative values for physical measurements or biologically impossible values, were removed from the dataset. This manual inspection and correction process ensured the removal of obvious errors that could negatively impact model training.

*Missing value handling:* The extent of missing values was assessed separately for phenotypic and genotypic datasets. For phenotypic data, missing values were minimal, representing less than 2% of the total data points. Given the random distribution of these missing values and their low overall proportion, we employed mean imputation. Specifically, missing values for a given feature were replaced with the mean value of that feature calculated across all samples within the same planting date and year. This approach was chosen to minimize bias, as the missingness was not concentrated in any particular group or feature. For genotypic data, represented as binary scores (presence/absence of a marker), missing values were coded as ‘0’, signifying the absence of the marker.

*Data transformation:* Phenotypic data, comprising continuous variables (e.g., plant height, leaf weight, awn length), were standardized using z-score normalization. This transformation ensures that all phenotypic features have a mean of 0 and a standard deviation of 1, preventing features with larger scales from disproportionately influencing the machine learning models. The formula for z-score normalization is: z = (x − μ)/σ, where x is the original value, μ is the mean of the feature, and σ is the standard deviation of the feature. Genotypic data, being binary, did not require normalization.

*Data type consistency:* We meticulously verified that all data types were consistent with their roles in the subsequent analyses. Phenotypic and disease severity data were confirmed as numeric and genotypic data were confirmed as binary. This step ensured compatibility with the chosen machine learning algorithms.

### Feature selection

Feature selection algorithms reduce the dimensions of the input data. Due to constraints coupled with selected or ignored features and subset sizes, feature selection algorithms try to find a subset of features that predicts the ML output well. Increasing prediction performance and providing faster and more cost-effective prediction performances are the main advantages of feature selection. Even if all features are applicable and contain information on the output variable, using all features can reduce the overall prediction performance. Although feature selection techniques are established methodologies in machine learning and have been previously used in plant science, their systematic and comparative application within the context of disease resistance prediction in barley, particularly using combined genotypic and phenotypic data, represents a distinct aspect of this study. In this research, we employed three distinct feature selection algorithms – RReliefF, Minimum Redundancy Maximum Relevance (MRMR), and F-test – to comprehensively identify the most informative features for predicting powdery mildew resistance in barley.The RReliefF algorithm is an extension of the Relief algorithm, designed for feature selection in ML tasks. It is beneficial for dealing with datasets that have noisy, incomplete, or multi-class data. The algorithm works by assigning weights to features based on their capability to discriminate between instances that are near to each other. Features that are more relevant for predicting the output variable receive higher weights. At the same time, irrelevant features receive lower weights, which can then be used to select a subset of features for model training^34^.

The Minimum Redundancy Maximum Relevance (MRMR) feature selection algorithm is used to identify a subset of highly relevant features. The selected features predict the output variable while being minimally redundant among themselves. The MRMR algorithm operates on mutual information, quantifying the information shared between features and the target variable and between the features themselves^35^.

The F-Test feature selection algorithm is a statistical method for selecting features with significant predictive power regarding the output variable. It is based on the F-statistic, which is derived from ANOVA tests. The F-test evaluates the degree of variance between groups compared to the variance within groups for each feature individually. The F-test assesses the null hypothesis that the means of the different groups are equal, implying that the feature does not discriminate well between the groups. A low F-value suggests that the feature does not contribute much to the group differences.

In contrast, a high F-value indicates that the feature is a good discriminator and, thus, potentially useful for prediction. However, the F-Test may overlook features only relevant in combination with others because it is a univariate method. Despite this limitation, it remains a popular choice for the initial screening of features to reduce dimensionality before applying more complex feature selection methods or training ML models.

### ML models

The DT, RF, NET, and GPR models are deployed in this paper. This Section presents a brief description of these models. The ML models are introduced more conceptually than mathematically. The mathematical explanations of the models can be found in textbooks^36,37^. These machine learning models are widely recognized and used in various fields, including plant science, but our study’s novelty lies in their specific and comprehensive application to predict powdery mildew resistance in barley using a novel combination of feature selection methods and integrated genotypic and phenotypic datasets. Furthermore, we employed Bayesian optimization to fine-tune the hyperparameters of these models, enhancing their predictive performance specifically for the research context.

DTs are a non-parametric supervised learning method for classification and regression tasks. Mathematically, a DT is a flowchart-like structure where each node denotes a “test” on a feature, each branch is the result of the test, and each node symbolizes a class label. The paths from root to leaf represent prediction rules. In a DT model, the data is split according to a specific criterion selected to maximize the homogeneity of the resulting subsets. The most common criterion for splitting used for trees, where the target variable is continuous, is variance reduction. The goal is to find the split that reduces the variance of the target variable within the subsets. The variance reduction for a split is given by

2 $$VarReduction=Var\left(S\right)-{\sum }_{v\in Values\left(A\right)}\frac{\left|{S}_{v}\right|}{\left|S\right|}Var\left({S}_{v}\right)$$

where Var(S) is the variance of the target variable in subset S. The DT algorithm recursively parses the data until it meets a stopping criterion. The resulting model provides an interpretable representation of the decision-making process. To mitigate overfitting, techniques such as pruning (removing parts of the tree that do not give power to classify instances), setting a maximum depth for the tree, or requiring a minimum number of samples to split a node can be used^38^.

The RF model is an ensemble learning method that operates by constructing many DTs at the training stage and outputting the class of the mean prediction of the individual trees in regression problems. Mathematically, an RF aggregates the predictions of several DTs to make more accurate and stable predictions than a single DT. An RF comprises many individual DTs that operate as an ensemble. The fundamental concept behind RF is a simple but powerful one. Many relatively uncorrelated models (trees) operating as a committee will outperform any individual constituent models. The low correlation between models is the key. So, the forest can perform well in a wide range of data sets, including those with high-dimensional feature spaces and complex data structures. In RFs, the tree construction process incorporates feature bagging (bootstrap aggregating), which involves randomly selecting a subset of features for consideration at each split point. This process de-correlates the trees by giving them different perspectives of the data to learn from, and hence, it ensures that the ensemble model does not overfit the training data. Mathematically, the prediction of an RF for a regression problem can be expressed as the average of the predictions of the N individual trees:

3 $$RF\left(x\right)=\frac{1}{N}{\sum }_{i=1}^{N}{T}_{i}\left(x\right)$$

where RF(x) is the prediction of the RF for input x, N is the number of trees, and ${T}_{i}\left(x\right)$ is the prediction of the ith tree. The RFs are robust against overfitting as they average out biases, can handle missing values, and are relatively unaffected by outliers, making them a powerful tool for a wide range of machine-learning tasks^39^.

The NETs represent a category of ML models that draw inspiration from the biological structure and operational mechanisms of the human brain. In the context of regression problems, NETs aim to predict continuous outcomes by learning complex mappings from inputs to outputs. The fundamental building block of a NET is the neuron, or node, which receives inputs, applies a weighted sum, and then typically passes the result through a non-linear activation function. A simple NET for regression can be represented as follows: The input layer consists of neurons corresponding to the dataset’s features. If there are n features, there will be n input neurons. One or more hidden layers can exist, each comprising many neurons. The output of each neuron in a hidden layer is a function of the weighted sum of its inputs plus a bias term:

4 $${h}_{ij}=f\left({\sum }_{k=1}^{n}{w}_{ijk}\cdot {x}_{k}+{b}_{ij}\right)$$

where ${h}_{ij}$ is the output of the jth neuron in the ith hidden layer, f is the activation function, ${w}_{ijk}$ are the weights, ${x}_{k}$ are the input features and ${b}_{ij}$ is the bias term^38^. The output layer produces the final prediction for the regression problem. In a single-output network, there is only one neuron in this layer, and its output is the predicted value:

5 $$y=f\left({\sum }_{j=1}^{m}{w}_{oj}\cdot {h}_{j}+{b}_{o}\right)$$

where y is the predicted output, ${w}_{oj}$ are the weights from the last hidden layer to the output neuron, ${h}_{j}$ are the outputs of the last hidden layer’s neurons, ${b}_{o}$ is the output neuron’s bias, and m is the number of neurons in the last hidden layer. The activation function f introduces non-linearity into the model, allowing the network to capture complex relationships. Common choices for f include the sigmoid, tanh, and ReLU functions. The training process for a NET entails the iterative adjustment of weights and biases. This is performed to minimize a predefined loss function, which quantifies the discrepancy between the model’s predicted outputs and the corresponding ground-truth values. Gradient descent, often with backpropagation, is a common optimization technique used to perform this adjustment iteratively. NETs can be extended to include multiple hidden layers (deep learning), various types of layers (like convolutional or recurrent layers), and different architectures tailored to specific types of regression problems. The flexibility and capacity of NETs to model non-linear relationships make them powerful tools for regression analysis^40^.

The GPR is a non-parametric, Bayesian approach to regression that is particularly powerful for modeling unknown functions and making predictions with an associated measure of uncertainty. The foundation of GPR lies in the assumption that the function values being predicted can be described by a Gaussian process—a collection of random variables, any finite number of which have a joint Gaussian distribution. Mathematically, a Gaussian process is fully specified by its mean function m(x) and covariance function $k\left(x.{x}{\prime}\right)$, also known as the kernel. The mean function represents the average prediction for the function at point x, and the covariance function encodes assumptions about the function’s smoothness and how points in the input space relate to each other. Given a set of observations $y$ at locations {X}, GPR infers the distribution over functions consistent with these observations. The predictive distribution for a new input ${x}_{*}$ is also Gaussian, with the mean and variance:

6 $$[\upmu \left({x}_{*}\right)={k}_{*}^{T}{\left(K+{\upsigma }_{n}^{2}I\right)}^{-1}y]$$

7 $$[{\upsigma }^{2}\left({x}_{*}\right)=k\left({x}_{*}.{x}_{*}\right)-{k}_{*}^{T}{\left(K+{\upsigma }_{n}^{2}I\right)}^{-1}{k}_{*}]$$

where ${k}_{*}$ is the vector of covariances between the new point and the training points, K is the covariance matrix of the training points, and the ${\sigma }_{n}^{2}$ is the noise variance. The GPR has several advantages, including uncertainty estimates for predictions and flexibility in choosing the kernel to encode prior beliefs about the function’s properties. However, it also faces computational challenges, particularly for large datasets, due to the inversion of the covariance matrix^41^.

although the individual machine learning models (Decision Tree, Random Forest, Neural Network, Gaussian Process Regression), feature selection algorithms (RReliefF, MRMR, F-test), and the Bayesian optimization approach for hyperparameter tuning employed in this study are established techniques, their specific integration and application within the context of powdery mildew resistance prediction in barley constitutes a novel methodological framework. Unlike previous studies that may have utilized subsets of these methods in isolation, our study systematically combines and compares a comprehensive suite of feature selection algorithms and machine learning models, optimized through Bayesian methods, to identify the most effective approach for predicting powdery mildew resistance from combined phenotypic and genotypic data. This holistic methodological approach, focused on comparative evaluation and optimized integration, differentiates this study and contributes to the advancement of machine learning applications in plant disease resistance research.

### Evaluation of the ML models

To statistically validate the performance differences between the models, we utilized Friedman’s test. Friedman’s test is a non-parametric statistical test suitable for comparing the performance of multiple models^42^. In this study, it was employed for pairwise comparisons of the ML models’ performance across different evaluation metrics. This study used 70% of the data for training and 30% for testing. Data validation was done in the training phase using five-fold cross-validation. Using RMSE, MAE, MSE, and R^2^ criteria, the model’s performance is evaluated in each training and testing stage.

8 $$MAE=\frac{1}{n}\sum_{i=1}^{n}\left|\frac{{y}_{i}-\overline{{y }_{i}}}{{y}_{i}}\right|$$

9 $$RMSE=\sqrt{\frac{1}{n}\sum_{i=1}^{n}{\left({y}_{i}-\overline{{y }_{i}}\right)}^{2}}$$

10 $${R}^{2}=1-\frac{\sum_{i=1}^{n}{\left({y}_{i}-\overline{{y }_{i}}\right)}^{2}}{\sum_{i=1}^{n}{\left({y}_{1}-{y}_{ave}\right)}^{2}}$$

In these equations, ${y}_{i} and \overline{{y }_{i}}$ are predicted value and actual value, ${y}_{ave}$ is the average of data set values, and *n* is the number of observations.

## Supplementary Information

Supplementary Information 1.

Supplementary Information 2.

Supplementary Information 3.

## Data Availability

All data generated or analysed during this study are included in this published article.

## Abbreviations

FS: Feature selection
GPR: Gaussian process regression
ML: Machine learning
MAE: Mean absolute error
NET: Neural networks
QTL: Quantitative trait loci
RF: Random forest
RMSE: Root mean squared error
DT: Decision tree
